# Probiotics in Cosmetic and Personal Care Products: Trends and Challenges

**DOI:** 10.3390/molecules26051249

**Published:** 2021-02-26

**Authors:** Scarlett Puebla-Barragan, Gregor Reid

**Affiliations:** 1 Centre for Human Microbiome and Probiotics, Lawson Health Research Institute, London, ON N6C 2R5, Canada; 2 Departments of Microbiology & Immunology and Surgery, University of Western Ontario, London, ON N6A 3K7, Canada; spueblab@uwo.ca

**Keywords:** probiotics, cosmetics, lysates, skin, microbiome, vaginal health

## Abstract

Probiotics, defined as “live microorganisms that, when administered in adequate amounts, confer a health benefit on the host,” are becoming increasingly popular and marketable. However, too many of the products currently labelled as probiotics fail to comply with the defining characteristics. In recent years, the cosmetic industry has increased the number of products classified as probiotics. While there are several potential applications for probiotics in personal care products, specifically for oral, skin, and intimate care, proper regulation of the labelling and marketing standards is still required to guarantee that consumers are indeed purchasing a probiotic product. This review explores the current market, regulatory aspects, and potential applications of probiotics in the personal care industry.

## 1. Introduction

According to the US Food and Drug Administration (FDA), a cosmetic is defined as “a product (excluding pure soap) intended to be applied to the human body for cleansing, beautifying, promoting attractiveness, or altering the appearance” [1]. This definition applies to products for skin, hair, and oral care. It is important to note that this description does not include any health claims.

The increased interest in microbes colonizing the human body, not simply those infecting it, has led to many studies attempting to manipulate the microbiome in a given niche, in favour of health. The use of beneficial microbes for this purpose has seen the field of probiotics grow substantially. Defined as “live microorganisms that, when administered in adequate amounts, confer a health benefit on the host,” probiotic applications range widely in type, scope, and application [2]. This includes cosmetic applications, where the market for probiotics is projected to grow at a 12% rate in the next ten years, with North America the driver [3].

This article will explore current research on probiotics for potential cosmetic and personal care applications and how “probiotic cosmetics” are currently being marketed. 

## 2. Cosmetics for Skin

The increase in products termed probiotic on the market does not necessarily equate with a reason to celebrate the successful translation of science to commerce and consumers. Too many products fail to comply with the characteristics required to be called probiotic. Many false claims and rampant misuse of the term has resulted in mainstream consumer channels providing incorrect information to consumers. Probiotics are not inside us, not in fermented food, not necessarily better if there are more species or a higher viable count. Formulations are being concocted not based on research evidence but on marketing and what might appeal to consumers. For example, products are being composed supposedly to improve vaginal health using strains not documented to compete with urogenital pathogens, improve immunity or do anything that can restore homeostasis to that region of the body. In other words, there are no data to support their selection. Yet, the internet, the use of words to reach the first page of search engines, and use of pseudo-experts for promotion provide a means for these products to be highly rated and appear to be the best clinically documented for preventing or curing bacterial or yeast infections in the vagina.

The net result is misleading and confusing to consumers, as well as making healthcare professionals leery of the whole field of probiotics. To counter this, we need to re-state important facts. 

For a product to be considered probiotic, it must comply with three core characteristics: 1. The strain(s) must be characterized, including genetically and phenotypically and a rationale given based on documented experiments published in peer-reviewed papers, for their inclusion for the intended use. 2. The product must contain sufficient live microorganisms at the time of use that are equivalent to when the product was shown in clinical studies to confer a benefit to the desired target site. 3. The delivery method, dosage, and duration of use should be based on scientific evidence in humans if humans are the intended recipient. 

It makes it difficult for potential users if the product label does not state strain designations because it becomes impossible to track the research performed on the contents. Dosages are rarely stated on labels, and some products only contain filtered extracts or ferments or lysed bacteria, meaning that no live microorganisms are present: thus, the product is not probiotic and the term should not have been used. 

The cosmetic industry has ventured into this space by focusing its efforts towards skincare. There are several areas of opportunity and great value in this concept. 

**Figure 1:**
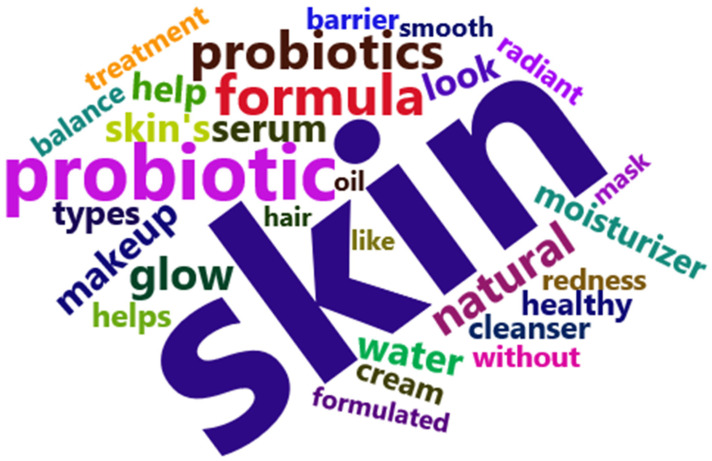
The top 30 words used in the claims of cosmetics marketed as probiotics. Word cloud generated using a compilation of the claims of 50 cosmetic products claiming to contain probiotics. Text analysis performed using the rtweet [6] and tm [7] packages in R version 4.0.2.

A search of the websites of two major retailers of cosmetics in North America revealed that at least 50 products are already being commercialized with a claim to contain probiotics [4,5]. Figure 1 shows a word cloud with the top 30 terms used in their statements. The majority of them are targeted for skincare, although some are for deodorants and hair care. The most common claims are geared towards “balancing” the skin microbiome, improving the skin barrier, and enhancing the skin’s overall appearance. Table 1 breaks down the types of products included in this analysis, and Table 2 includes the ingredients as listed on their labels; all of them correspond to different types of products targeted for skincare. All products included in this analysis can be used by any gender. 

These claims by themselves require critiquing. There is no single healthy skin microbiome, so what would it take to ”balance” the one that a given individual possessed? Furthermore, the skin has many layers, with microbes being detected in the dermis, adipose, follicle, epidermis [8]. A product claiming to “balance” the microbiome should have studies indicating in many subjects, preferably hundreds, how a given probiotic product changes the various layers of microbiota in such a way as to restore and maintain it to what is deemed healthy for each individual. Since it seems highly unlikely that such studies have been performed for most if any products, claims of balancing the skin microbiome should not be made. This is important because consumers like the sound of products that do that, especially since terms like the “microbiome” and “balance” are being used so widely.

Enhancement of the skin’s overall appearance can be subjective for consumers, but it also has some scientific principles. Factors such as the reduction of the contrast, presence of visible ageing marks or spots, skin colour, melanin, and hemoglobin can be measured [9]. This and other assessments provide the means to give tangible results that can then provide substance to claims of improvements.

The ability of certain probiotic strains to improve epithelial and epidermal barrier function has been reported. The latter is so critical in the function of the skin and a well-used target for making claims that appeal to consumers. If strains being used as cosmetics do indeed improve barrier function, experiments can be performed to verify this. Indeed, researchers from a well-renowned cosmetic company have shown that a lysate from the probiotic *Bifidobacterium longum* reuter strain could decrease vasodilation, edema, mast cell degranulation, and TNF-alpha release, and using trans-epidermal water loss to assess barrier function, showed improvement with application of the lysate containing product [10]. Some products state that they include a filtrate of either ferments or lysates. In the case of filtrates, bacterial cells (alive or not) are removed along with potentially some other larger weight molecules (e.g., peptides). This might remove some of the bioactive compounds of the preparation and components of the bacterial cells required to observe specific benefits. Therefore, filtrates are excluded from the definition of postbiotics and cannot be deemed to be probiotic.

Lysates are cells whose outer membrane has been broken down due to chemical or physical processes [11]. These preparations have been used in medical practice as immunomodulators for fifty years. They can contain bacterial components that up-regulate the immune response of the host cells; they are particularly effective in the management of infections of the respiratory tract [12]. Lysates of *Lacticaseibacillus rhamnosus* GG and *B. longum* can increase tight-junction barrier resistance *in vitro* by modulating protein components [13]. Although there is value in using these types of preparations, further studies are still required on a strain-dependent basis before drawing conclusions and making claims. The cell composition, elasticity, and activation of macrophages differ between bacterial strains, even within the same species [14]. In one study, lactobacilli lysates altered their ability to increase re-epithelialization of keratinocytes [15], again emphasizing the need to check strain properties prior to making claims.

Intriguingly, little has been reported on the chemical composition of the lysates being used in cosmetics. This should require cell wall and analysis, including the use of liquid chromatography–tandem mass spectrometry (LC-MS/MS)-based metabolomics [16] or newer methods such as surface-enhanced Raman spectroscopy (SERS) [17]. In doing so, it will soon be extremely apparent that metabolite types and quantities differ between strains, and therefore their application to human tissues would also differ. This again illustrates the need to perform tests in humans with whole microbial cells, lysates, or filtrates to show what activity is being promoted by the application of any given product; ideally to know which component of the lysate is responsible.

There are numerous studies providing evidence of the benefits of specific probiotic strains for skin health [18,19,20]. In addition, the mechanisms of anti-ageing suggest strains can help to regulate pH, reduce oxidative stress, protect from photoaging, and improve the skin barrier function [21].

However, the cosmetic industry needs to be consistent and transparent in its labelling practices and direct efforts to generating more scientific evidence before making claims.

## 3. Topical Delivery and Formulation of Probiotics 

Not all applications for skincare for males or females require local application. Orally administered probiotics have been demonstrated to affect the intestinal microbiome leading to a potential improvement in skin conditions such as atopic dermatitis, acne, or rosacea [22,23]. Early studies suggested that probiotic use during gestation and early life may be required to reduce the incidence and adversity of atopic dermatitis [24], implying immune modulation and improving the maturing gut barrier function [25].

Freeze-drying of probiotic strains is commonplace. However, depending on the drying protectant used, final viability can vary. The most used protectants are skim milk, serum, trehalose, glycerol, betaine, adonitol, sucrose, glucose, lactose, and polyethylene glycol; these may not be compatible with the intended use of the product or the physicochemical characteristics of the formula [26]. When using this method, the strains should not be exposed to water, otherwise they will prematurely rehydrate [27].

Microencapsulation is used to extend the shelf-life and viability of probiotics. It is primarily to ensure that organisms resist the digestive system’s extreme environment [28], but it has been used in topical formulas [29]. Most commonly, the microbes are embedded in a protective matrix of biopolymers or lipids.

It is challenging for the cosmetic industry to create topical formulas that retain probiotic bacterial viability from production to the value chain and onto the consumer. Moisture would allow the dried organisms to hydrate and multiply or die, so oil-based formulations are needed. The question becomes how easily the organisms can emerge from the oil once placed on the skin, and thence become metabolically active sufficient to deliver the probiotic effects required.

Many creams are not produced in sterile conditions, therefore preservatives are often added with bactericidal and/or bacteriostatic effects. These potentially can not only affect the probiotic strain viability but also inadvertently alter the microbiota of the recipient.

Regulation of probiotics is primarily concerned with safety. There is no specific requirement for commercializing probiotics, and products are regulated according to their final use, whether it is as a drug, medical device, food, dietary supplement, or cosmetic. The descriptiveness and level of documentation required to claim a cosmetic probiotic is substantially less than for one making drug claims in Canada and elsewhere [30]. Unfortunately, in a bid to maximize profit, some companies make disease or illness-alleviation statements associated with their cosmetic products, when this should be reserved for drugs or clinically proven supplements.

Due to safety concerns, cosmetic products are expected to have a low content of microorganisms (below 500 colony forming units (CFU)/g for eye-area products and 1000 CFU/g for the rest) [31]. It is not a viable option for them to contain live bacteria, meaning that there cannot be a cosmetic that is a true probiotic. However, they can still contain components sourced from probiotic strains that could be beneficial. These can constitute bacterial lysates, ferments, and filtrates, sometimes referred to as postbiotics, defined as a “preparation of inanimate microorganisms and/or their components that confers a health benefit on the target host. [32]”. This definition does not include purified metabolites or components without cells, which should instead be listed following their chemical nomenclature. Filtrates without cell components are not considered postbiotics. However, bacterial lysates and ferments might fit into this category depending on their composition.

Biochemically, fermentation is an anaerobic metabolic process where carbohydrates (for example, lactose) are partially oxidized to generate energy for the cell, with lactic acid produced as a result. Fermented foods and beverages are defined as “foods made through desired microbial growth and enzymatic conversions of food components [33]. This should not be confused with the ferments for skin application. In that case, the process of fermentation has taken place but not with respect to using or converting human food. Fermentation can be either an aerobic or anaerobic process in which a living organism (or its enzymes) chemically modify a substrate to generate a product of interest [34]. Therefore, if ferments included in a cosmetic product contain viable probiotic strains, and if they remain live until they reach the host target site, these products could potentially be marketed as probiotics.

**Figure 2:**
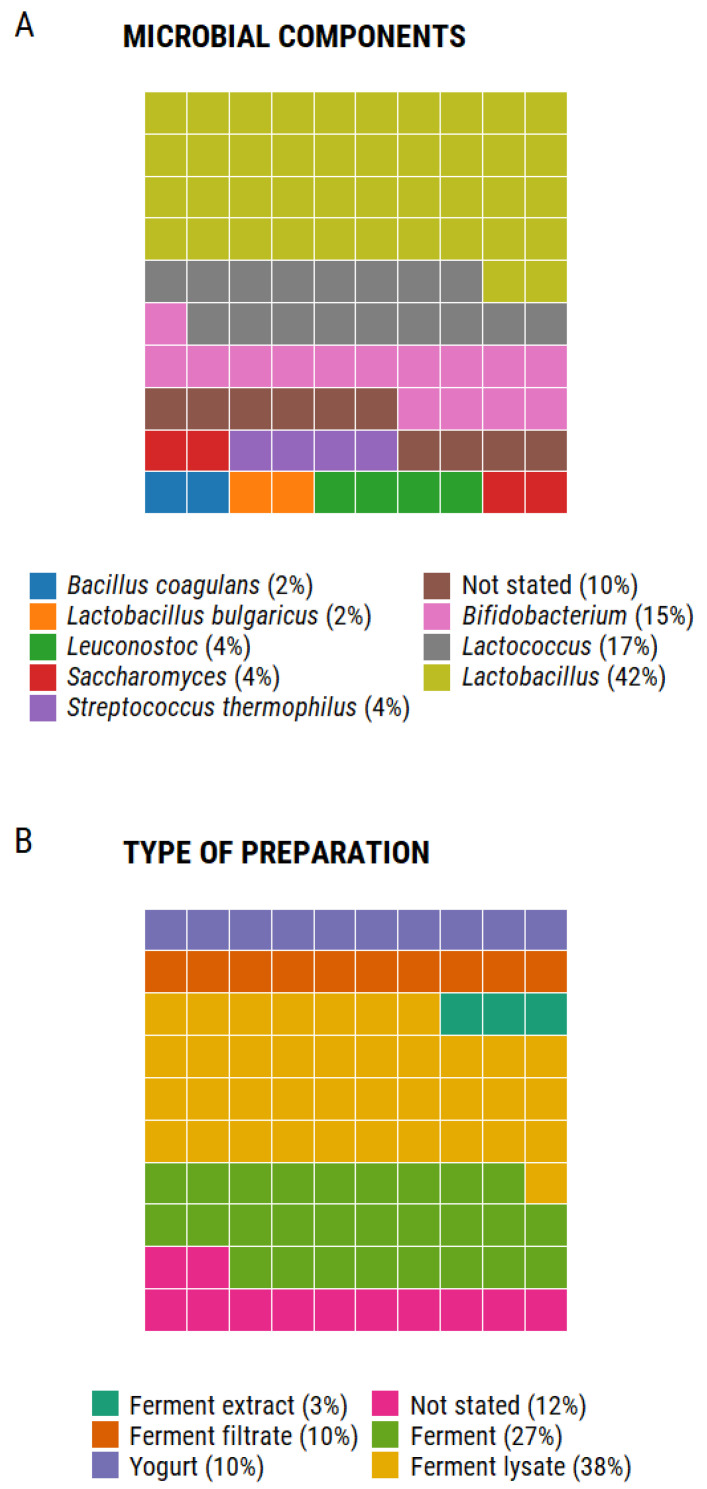
Debrief of the ingredients listed in 50 cosmetic products. A total of 71 items listed in the ingredients label of the products were included in this analysis; waffle charts depict the proportion of each component as parts of a whole [40]. Panel A shows the frequency in which each microbial component was included. Panel B shows the frequency in which each type of preparation was used in the products analyzed. Generated using R version 4.0.2. Percentages were rounded to the nearest whole number for ease of visualization.

Unfortunately, as shown in Figure 2, from the ingredients listed in the labels of the 50 cosmetic products we analyzed (Table 2), none of them stated the strain designation of the microorganism included, and only 8% of the listed ingredients included the name of the species used. Those products listing the use of *Bifidobacterium* included it on their labels as “Bifida ferment lysate,” documented as an ultrasound inactivated suspension of *Bifidobacterium longum* reuter in aqueous medium [10], which as previously described, can improve barrier function as well as decrease skin sensitivity. Nonetheless, products containing this ingredient cannot be marketed as probiotics due to the absence of live bacteria.

In terms of the type of preparation, 78% of the ingredients listed on the labels corresponded to ferments, either as extracts, filtrates, or lysates. Yogurt is listed in 10% of the ingredients, however, there is no information about the type of bacteria or substrate, nor is it indicated if it contains live bacteria. Finally, 12% of the products do not indicate the type of preparation in which the microbial ingredients were included. Overall, the information is vague and does not allow for a critical analysis of the potential probiotic or postbiotic characteristics of the formula.

With proper regulations in place, and potentially labelled as over-the-counter (OTC) drugs instead of cosmetics, there is value in the use of probiotics as topical treatments. Particularly, their antibacterial and immunomodulatory properties make them promising candidates to target skin ailments such as acne, psoriasis, and atopic dermatitis, as well as to aid in wound healing [35,36,37,38,39]. Nonetheless, further research in humans and randomized clinical trials are still required to validate these potential uses.

## 4. Probiotics for Female Intimate Care 

A healthy vaginal environment is in most cases populated by an abundance of lactobacilli. Various triggers, from the use of douches and antibiotics to multiple sexual partners and influx of pathogens into the area, disrupt the homeostasis giving rise to bacterial vaginosis, urinary tract infections, candidiasis, and other conditions. This provided a rationale 48 years ago to supplement the urogenital tract with lactobacilli to restore a healthy state [29,41]. Since then, the vaginal administration of probiotic strains of *Lactobacillus* through suppositories or vaginal ovules has been explored [23].

Beginning with instilling *Lacticaseibacillus* (formerly, *Lactobacillus*) *rhamnosus* GR-1 into the vagina [42], a range of strains have been tested, including *Limosilactobacillus* (formerly *Lactobacillus*) reuteri RC-14 and *Lactobacillus crispatus* CTV05 to reduce the recurrence of urinary tract infection (UTI) [43,44], *Lacticaseibacillus rhamnosus* IMC 501 in combination with *Lactobacillus paracasei* IMC 502 to maintain vaginal homeostasis [45] and *L. rhamnosus* Lcr35 for BV and vulvovaginal candidiasis [46,47].

Given the significant negative impact of antimicrobial therapy on the urogenital microbiota and failure to restore homeostasis, probiotic strains have been used in combination to help with recovery. These include *Lactobacillus gasseri* EN-153471 (EB01) for the management of BV [48] and *L. rhamnosus* GR-1 plus *L. reuteri* RC-14 in combination with antibiotics or antifungals [49,50,51]. Additional strains have become available in the American market with minimal clinical and scientific documentation [52].

The supplements are believed to function through ascension from the rectal skin to the vagina, where they reduce pathogen ascension and inhibit and displace pathogens while also conferring antimicrobial defences through the production of bioactive compounds such as lactic acid, hydrogen peroxide, and bacteriocins.

Therefore, these are essentially cosmetic in action on the skin, but they are promoted through a higher level of regulation where some functional and structural, or even disease risk reduction claims can be made. The application of strains directly into the vagina using suppositories that are applied vaginally is approved in Canada [53]. Other products are being delivered through coating tampons and pomades [54,55,56], but further evidence is required to confirm they are probiotic and benefit the host.

An emerging area is for probiotic strains to reduce urogenital malodor that significantly impacts the quality of life of women, especially in combination with bacterial vaginosis [57]. Many non-probiotic products such as vaginal douches, vinegar rinses, and fragrances claim to help reduce malodor. However, their efficacy is dubious, and they can increase the risk of infection, including sexual acquisition through disruption of the beneficial microbes [58,59]. An advantage of an effective probiotic would come from its ability to grow and produce metabolites that degrade or neutralize malodorous compounds [60,61]. Depending on the nature of such a product, it may have to be registered as an OTC drug and not as a cosmetic or personal care product.

The development of topical gel containing probiotic lactobacilli is already underway, with promising results for treating vulvo-vaginal candidiasis [62].

It could be argued that applications in the urogenital tract do not strictly fall within the definition of a cosmetic, namely intended to restore or improve a person’s appearance. Likewise, applications for reducing halitosis [63,64,65] may also not fit, depending on how ‘appearance’ is defined and interpreted. Whereas reducing acne symptoms with lactobacilli in a topical cream would fit as a cosmetic [66].

## 5. Conclusions 

The recognition that certain types of microbes provide health benefits to the host and that the human body and planet are literally filled with microbes, has brought new opportunities to the management of personal and ecosystem health. Companies in all spheres of business, including cosmetics, have taken advantage of this knowledge to develop new products and increase profits. Terms such as probiotics, prebiotics, and microbiome were unheard of in cosmetic products a mere twenty years ago. Their use would be encouraging if it coincided with strong scientific research supporting claims and uncovering the mechanisms of action of the strains and material being promoted. Unfortunately, this is rarely the case.

While chemistry is a mainstay of the cosmetic field, it has not been sufficiently well applied to identify the molecules responsible for the benefits provided by microbial products. Given the expansion of the microbiome field, an emergence of microbiology and chemistry expertise will be needed to ensure high-quality cosmetics adherent to definitions (of a probiotic, prebiotic, etc.) are able to reach consumers.

There is no question that the modulation of microbes can lead to novel ways to improve appearance and well-being. This will provide regulatory challenges as it brings cosmetic products into the health realm. While advocating the need for regulatory agencies to upgrade their often-antiquated systems and categories, we need to insist on product safety, clinical verification, and proof of using high standards for handling, storing and applying products containing microbes and their metabolites or cell walls. Unproven claims help no-one, whereas good scientific investigation can bring forth products of great merit to human health and well-being.

## Figures and Tables

**Table 1:** Cosmetic products currently marketed as probiotics. Percentage corresponding to the different types of products currently marketed as “probiotic cosmetics.” Data obtained from 50 products advertised by major retailers of cosmetics [4,5].

TYPE OF PRODUCT	PROPORTION OF PRODUCTS
Deodorant	2%
Primer	2%
Balm	4%
Soap bar	4%
Foundation	6%
Cleanser	10%
Exfoliant	10%
Gel	10%
Mask	12%
Serum	16%
Cream	24%

**Table 2:** Cosmetic products and their ingredients. Data obtained from 50 products advertised by major retailers of cosmetics. * The ingredient listed as “Bifida ferment lysate” corresponds to a lysate from *Bifidobacterium longum* reuter.

PRODUCT ID	TYPE OF PRODUCT	LIST OF INGREDIENTS
1	Balm	*Lactococcus* ferment lysate
2	Balm	*Lactobacillus* ferment
3	Cleanser	Bifida ferment lysate *
4	Cleanser	*Lactobacillus* ferment
5	Cleanser	*Lactobacillus* ferment
6	Cleanser	Bifida ferment lysate *
7	Cleanser	*Lactobacillus* ferment
8	Cream	*Lactobacillus* ferment, *Lactococcus* ferment lysate, Bifida ferment lysate *, *Lactobacillus*, *Streptococcus thermophilus* ferment
9	Cream	*Lactobacillus* ferment
10	Cream	*Lactobacillus* ferment
11	Cream	*Lactobacillus* ferment
12	Cream	Bifida ferment lysate *
13	Cream	*Lactobacillus* ferment
14	Cream	*Bacillus coagulans*
15	Cream	*Lactococcus* ferment lysate
16	Cream	*Lactobacillus* ferment
17	Cream	*Lactobacillus* ferment lysate
18	Cream	*Lactobacillus* ferment
19	Cream	Bifida ferment lysate *
20	Deodorant	*Saccharomyces* ferment filtrate
21	Foundation	*Lactobacillus* ferment
22	Foundation	*Lactobacillus*
23	Foundation	*Lactococcus* ferment lysate
24	Gel	*Lactococcus* ferment lysate
25	Gel	*Lactobacillus*, *Lactococcus* ferment extract
26	Gel	*Leuconostoc* ferment filtrate
27	Gel	*Lactococcus* ferment lysate
28	Gel	*Lactobacillus*, Greek yogurt, yogurt, yogurt powder
29	Mask	*Lactobacillus*, Greek yogurt, yogurt, yogurt powder
30	Mask	Bifida ferment lysate *
31	Mask	*Lactococcus* ferment lysate
32	Mask	*Lactococcus* ferment lysate
33	Mask	*Lactobacillus* ferment
34	Mask	*Lactobacillus* ferment, *Lactococcus* ferment lysate, Bifida ferment lysate *, *Lactobacillus*, *Streptococcus thermophilus* ferment
35	Exfoliant	*Lactobacillus* ferment
36	Exfoliant	*Lactococcus* ferment lysate
37	Primer	*Saccharomyces* ferment filtrate
38	Exfoliant	*Lactobacillus* ferment lysate, *Leuconostoc* ferment filtrate
39	Exfoliant	*Saccharomyces* ferment filtrate
40	Exfoliant	*Lactobacillus* ferment lysate, *Leuconostoc* ferment filtrate
41	Serum	*Lactococcus* ferment lysate
42	Serum	*Lactobacillus ferment*, *Lactococcus* ferment lysate, Bifida ferment lysate *, *Lactobacillus*, *Streptococcus thermophilus* ferment
43	Serum	*Lactobacillus bulgaricus* ferment filtrate
44	Serum	Bifida ferment lysate *
45	Serum	*Lactobacillus* ferment extract
46	Serum	Bifida ferment lysate *
47	Serum	*Lactobacillus*
48	Serum	Bifida ferment lysate *
49	Soap bar	Bifida ferment lysate *
50	Soap bar	Yogurt
